# Long and Short-Term Effects of Hypothermic Machine Perfusion vs. Cold Storage on Transplanted Kidneys from Expanded Criteria Donors—A Matched Comparison Study

**DOI:** 10.3390/jcm12175531

**Published:** 2023-08-25

**Authors:** Matthias Axelsson, Per Lindnér, Nils-Gunnar Pehrsson, Seema Baid-Agrawal

**Affiliations:** 1 Transplant Institute, Sahlgrenska Academy at the University of Gothenburg and Sahlgrenska University Hospital, 41345 Gothenburg, Sweden; per.lindner@vgregion.se; 2 Statistiska Konsultgruppen, 41463 Gothenburg, Sweden; info@stat-grp.se

**Keywords:** kidney transplantation, hypothermic machine perfusion, static cold storage, delayed graft function, graft survival, graft function, expanded criteria donor

## Abstract

Hypothermic machine perfusion (HMP) has been shown to reduce delayed graft function (DGF)-rates in kidneys from expanded criteria donors (ECD) and may increase graft survival compared with static cold storage (SCS). This single-center, retrospective observational study aimed to evaluate this effect. The primary endpoint was the DGF-rate, defined as the use of dialysis in the first postoperative week, excluding the first 24 h. The main secondary endpoint was graft survival at 5 years. Recipients of ECD-kidneys between 2013 and 2021 with ≤2 grafts were included (n = 438). The SCS-kidneys were marginal-matched by propensity score to the HMP-group for donor age, cold ischemia time, and graft number. Multivariable adjusted analysis for confounders in the unmatched cohort and caliper-based ID-matching constituted sensitivity analyses. HMP showed a trend to lower DGF-rate in the marginal-matched comparison (9.2% vs. 16.1%, *p* = 0.063). This was strengthened by a significant benefit observed for HMP in both the sensitivity analyses: an adjusted OR of 0.45 (95% CI: 0.24; 0.84; *p* = 0.012) in the multivariable analysis and DGF-rate of 8.7% vs. 17.4% (*p* = 0.024) after ID-matching. The 5-year graft survival rate was >90% in both groups, with no benefit using HMP (HR = 0.79; 95% CI:0.39–1.16; *p* = 0.52). Our results suggest that HMP may be effective in decreasing DGF-rates, however, without any significant benefit in graft survival.

## 1. Introduction

The persistent shortage of donor kidneys has led to increased utility of kidneys from expanded criteria donors (ECD). Despite an elevated risk of delayed graft function (DGF) and a 70% increased risk of graft failure compared to recipients of standard criteria donor (SCD) kidneys [1,2], recipients of the ECD kidneys have an increased survival rate as compared to being on dialysis. ECD-grafts translate into poorer long-term graft and patient survival rates as compared to SCD grafts, with 5-year graft survival of 49–80% according to a recent overview, although this varies greatly between centers [3,4]. These grafts are also more susceptible to ischemic-reperfusion. Therefore, limiting injury is important Static cold storage (SCS) was developed during the early years of transplantation, and is the gold standard for organ preservation [5,6]. The University of Wisconsin (UW) solution was later introduced and proven to be effective due to its cell impermeant agents that prevent the cells from swelling during cold ischemic storage. Even though 20–30% of kidneys do not function immediately after transplantation, organ preservation has been mainly unchanged until the introduction of hypothermic machine perfusion (HMP), developed as an improvement to SCS. During HMP, a pump is connected to the major vessels of the graft, perfusing it with a protective solution. HMP is standard at many centers for ECD kidneys owing to its well-established advantage over SCS, as a landmark randomized controlled trial first suggested in 2009 [7]. In this study, HMP significantly decreased the risk of DGF and increased 1- and 3-year graft survival in deceased donor kidneys compared with SCS [8,9]. The graft survival benefit was even more pronounced in ECD kidneys (3-year 86% for HMP vs. 76% for SCS) [7,9]. These results have since been validated to varying degrees by multiple studies, most consistently in terms of reducing DGF-rates [4,10]. However, there is a paucity of sound evidence on the effect of HMP on long-term graft survival and function [10].

During SCS and HMP alike the kidneys sustain cold ischemic injury, and increased cold ischemia time (CIT) is an established risk factor for developing DGF and decreased graft survival [11]. Therefore, a current challenge for transplant centers is to limit the CIT. HMP has also been shown to be most effective for kidneys developing DGF, and research is aimed at identifying these kidneys at risk for DGF prior to transplantation [8]. Donations after cardiac death (DCD) are in many regions the fastest growing donor pool, and increasingly a subject of research trying to alleviate their high risk of DGF. Lately, promising research has been published investigating alterations to the preservation technique, limiting the CIT by either oxygenating the perfusate or perfusing the kidney at normothermic temperatures [12,13]. Whether these methods are effective in ECD-kidney transplantation has not yet been thoroughly investigated.

Death-censored graft survival rates in Sweden are reportedly on the higher end, consistently > 90% at 5 years for kidneys from deceased donors [14,15]. HMP has been used at Sahlgrenska University Hospital (SU) since 2014 for ECD kidneys, but no study from our center has been conducted to date to evaluate the results. There are also limited reports on HMP efficacy in populations with relatively high graft survival rates. This exploratory study aimed to investigate whether HMP is better than SCS in preventing DGF and improving graft survival in ECD kidney transplantation performed at SU.

## 2. Materials and Methods

### 2.1. Population and Data Collection Procedures

This matched retrospective single-center study included patients who received ECD kidney transplants at SU between 2013 and 2021. ECD was defined as a brain-dead donor aged ≥ 60 years or ≥50 years with at least two risk factors: history of hypertension, cerebrovascular cause of death, and final serum or plasma creatinine ≥ 132 µmol/L [1]. Recipients with more than two kidney grafts were excluded from the study. Recipients of multiple organs at the time of transplantation were not excluded as their numbers were expected to be limited. AB0-matching was required for ECD-kidney transplantation, which resulted in no AB0-mismatches. The recipients were categorized according to the method of preservation, HMP, or SCS, and endpoints were compared between the groups. Follow-up data were collected until October 2022. Data were collected from our local transplantation registry, donor characteristics were completed using data from Scandiatransplant’s registry YASWA, and survival data from the National Swedish Renal Registry (SNR). Creatinine values and other missing data were collected from patient charts. Cold ischemia time (CIT) was defined as the time between the discontinuation of perfusion in the donor and the start of reperfusion in the recipient. Donor terminal serum/plasma-creatinine and the terminal estimated glomerular filtration rate (eGFR) based on these creatinine values were the last values before graft allocation. Kidney Donor Profile Index (KDPI) is a simple way to appreciate the likelihood of graft loss in kidneys from deceased donors by combining a variety of donor factors into a single number expressed as a percentage. To calculate the KDPI, we first calculated the donor’s Kidney Donor Risk Index (KDRI) using only donor factors as described by Rao et al. [16]. The KDPI was then allocated by cross-matching the KDRI to mapping tables for the respective years of transplantation, provided by the Organ Procurement and Transplantation Network and available on their website [17]. As race is not recorded in Sweden, donor race was assumed to be non-black for all donors because of the presumed very limited number of black donors, the population of Sweden being predominantly white. We expect this to have minimal impact on the final KDPI of the groups because of the already expected high KDPI [18]. Similarly, even for calculating the eGFR, the recipient race was considered non-black. We chose to calculate eGFR using the chronic kidney disease epidemiology collaboration (CKD-EPI) 2009 equation using a race coefficient since a recent large study on a Swedish population revealed a greater bias using the new 2021 race-free equation [19,20].

The grafts were thoroughly flushed during allocation using the Institut Georges Lopez-1 (IGL-1) solution. LifePort^®^ (Organ Recovery Systems; Itasca, IL, USA) was used for machine perfusion with the manufacturer’s standard settings. The preservation solutions were Kidney Perfusion Solution (KPS-1) or Beltzer MPS for HMP, and Static Preservation Solution (SPS-1) for SCS. The decision to use HMP or SCS was based on the fulfillment of ECD criteria and logistical factors. Kidneys from ECD were preserved using HMP. There were routinely two exceptions where ECD kidneys received SCS instead of HMP; kidneys imported from other transplant centers not using HMP or simultaneous heart donation from long-distance centers. However, owing to anatomical variations, time constraints, and logistical challenges, we expected a relatively low adherence to these routines and a significant use of SCS even for ECD kidneys.

The standard induction therapy before transplantation consisted of basiliximab and methylprednisolone. Standard maintenance immunosuppression post-transplantation is comprised of triple therapy with steroids, mycophenolic acid, and calcineurin inhibitors.

### 2.2. Endpoints

The primary endpoint was the DGF-rate; DGF was defined as the need for dialysis in the first postoperative week, excluding the first 24 h (Table 1). The secondary endpoints and their definitions are presented in Table 1. For survival analyses, the last dates of patient observation and observed graft function were defined as the latest follow-up date and the date of the last serum or plasma creatinine level, respectively.

### 2.3. Statistical Analysis

The following important predictors were presumed to differ between the two groups at baseline and constituted the basis for matching: donor age, cold ischemia time (CIT), and the number of previous grafts. To compare the two groups, three methods were used: the best marginal distribution matched group as the primary analysis, multivariable logistic regression adjusted for multiple confounders in the entire (unmatched) cohort as the first sensitivity analysis, and the best caliper-based ID matched group as the second sensitivity analysis. Unadjusted analyses of all the subjects constituted complementary analyses. For further information about each matching method and the parameters used, please refer to the Matching Methods section in Appendix A. The outcome variables were not available until the matching procedures were performed and evaluated to ensure blinding. The matching quality of each variable was evaluated using the standardized mean difference (SMD), calculated as the mean difference between groups divided by the pooled standard deviation (SD) of the groups. An SMD < 0.2 was regarded as good and <0.1 very good [21]. The best-matched group according to these baseline factors and including the greatest number of participants in each group, was used in the primary analysis, and the other two were used for sensitivity analyses.

Unadjusted comparisons were performed using Fisher’s exact test for dichotomous variables, Fisher’s non-parametric permutation test for continuous variables, and the Chi-square test for non-ordered categorical variables. Patient survival data were calculated using the Kaplan–Meier survival analysis method, log-rank test, and Cox proportional hazard regression. To correctly adjust for death as a competing event to graft loss in graft survival analysis, we used the cumulative incidence function, Gray’s test and Cox proportional hazards regression model [22,23]. Graft survival rates were calculated as 1 subtracted by the cumulative incidence of graft loss at 1, 3, and 5 years. Adjusted comparisons between the groups were performed using multivariable logistic regression for binary variables and analysis of covariance (ANCOVA) for continuous dependent variables. For paired analyses after caliper matching, Fisher’s paired non-parametric permutation test was used for continuous variables and the Sign test for dichotomous variables.

Continuous variables are presented as mean, standard deviation (SD), median, and range. Categorical variables were presented as numbers and percentages. All analyses were performed using SAS^®^ v9.4 (SAS Institute Inc., Cary, NC, USA). All significance tests were two-sided and were conducted at a 5% significance level.

### 2.4. Ethics

The study was approved by the Swedish Ethical Review Authority (D.nr. 2022-02644-01).

## 3. Results

### 3.1. Matching Results

**Figure 1:**
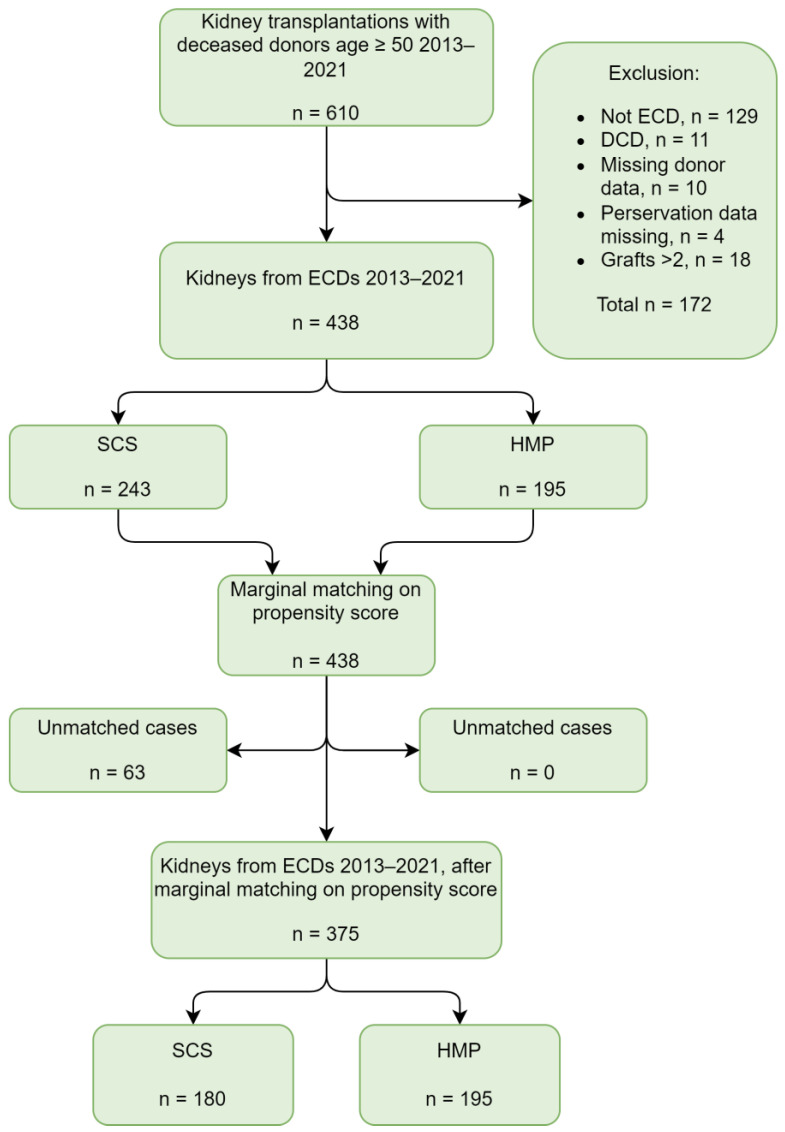
Study population: Inclusion, exclusion, and matching. ECD = expanded criteria donor, DCD = donation after cardiac death, SCS = static cold storage, HMP = hypothermic machine perfusion.

A total of 381 kidneys from ECD donors were transplanted between 2014 and 2021, excluding recipients of ≥2 grafts. A significant number of SCS controls was lost after our first round of matching regardless of the matching method; the best-matched SCS control group included only 136 participants. Therefore, SCS kidneys from 2013 were included to increase the pool, resulting in a final total study population of 438, of which 243 were SCS and 195 were HMP kidneys (Figure 1, Table 2). Marginal distribution matching on the propensity score generated the best-matched groups according to our criteria, resulting in 180 SCS and 195 HMP kidneys in the primary analysis (Table 3). Except for donor terminal creatinine and eGFR, SMD was <0.2 for every donor and recipient baseline characteristic, and no significant difference was observed between the groups.

The best ID-matching for the sensitivity analysis was caliper-matching with a caliper width of 8 years for donor age, 1.5 h for CIT, and exact matching for graft number, resulting in 172 subjects from each group (Table A1 in Appendix A).

### 3.2. Baseline Characteristics

The donor groups were similar in terms of baseline characteristics, except for donor age (68.5 years in the HMP vs. 65.7 in the SCS group, *p* = 0.0002) and CIT (14.4 h vs. 12.8 h, *p* < 0.0001), which were included as matching parameters, as well as KDPI (87.8% vs. 83.6%, *p* = 0.0003), terminal serum/plasma creatinine (92.6 vs. 79.7 µmol/L, *p* = 0.004) and terminal eGFR (74.3 vs. 82.2 mL/min/1.73 m^2^, *p* = 0.0006) (Table 2). The difference in recipient age was borderline significant (60.9 years vs. 59.0 years, *p* = 0.052). Duration of dialysis, frequencies of comorbidities and kidney disease, and immunological profiles were comparable between the groups, having an SMD < 0.2. After marginal-matching every recipient variable achieved an SMD < 0.2, ensuring comparability between the groups on the collected variables, and there were no significant differences between them (Table 3). This was also true for the caliper-based matching for sensitivity analysis (Table A1), although the donor KDPI did not reach an SMD of <0.2 (87.2 vs. 84.9, *p* = 0.056).

### 3.3. Delayed Graft Function (DGF)

Our primary marginal-matched analysis revealed a small trend for a lower DGF-rate of 9.2% in the HMP group versus 16.1% in the SCS group (Table 4); however, the difference did not reach statistical significance (mean difference: −6.9 [−14.1; 0.4], *p* = 0.063).

The trend was strengthened by significant reductions in DGF-rates in the two sensitivity analyses. The first sensitivity analysis, the logistic regression analysis in the whole cohort (prior to matching), modeled for the risk of the DGF and adjusted for donor and recipient age, CIT, graft number, donor terminal creatinine and eGFR, revealed that HMP reduced the odds of DGF by 55% (adjusted OR = 0.45; 95% CI:0.24–0.84; *p* = 0.012) (Table 5). In the second sensitivity analysis, after ID-matching, the DGF-rate was 8.7% in the HMP group versus 17.4% in the SCS group (*p* = 0.024, Table A2 in Appendix A). The paired analysis of the ID-matched subjects reported 14.0% of pairs in which the SCS kidney had DGF when the HMP did not, whereas in only 5.2% of pairs, the outcome was reversed (*p* = 0.014, Table A3 in Appendix A). Univariate differences of the outcome variables of the sensitivity and complementary analyses are available in Table A2 and Table A4 in Appendix A.

There was no significant difference in median DGF-duration (8 vs. 9 days) nor in primary non-function rates (0.5% vs. 1.1%). Mean DGF-duration was longer in the SCS-group, sensitivity analyses included, due to an outliner whose graft started functioning after 330 days.

### 3.4. Survival Analysis

**Figure 2:**
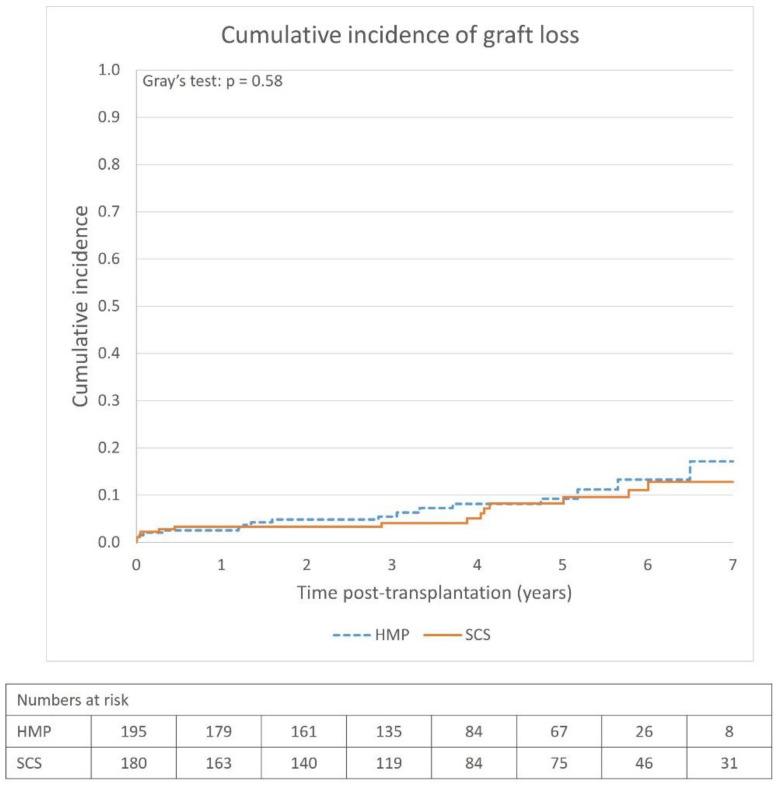
Post-transplantation cumulative incidence of graft loss in HMP and SCS groups, marginal-matched (n = 375) and adjusted for death as competing risk. Numbers of grafts lost: HMP = 17; SCS = 14. HMP = hypothermic machine perfusion, SCS = static cold storage.

**Figure 3:**
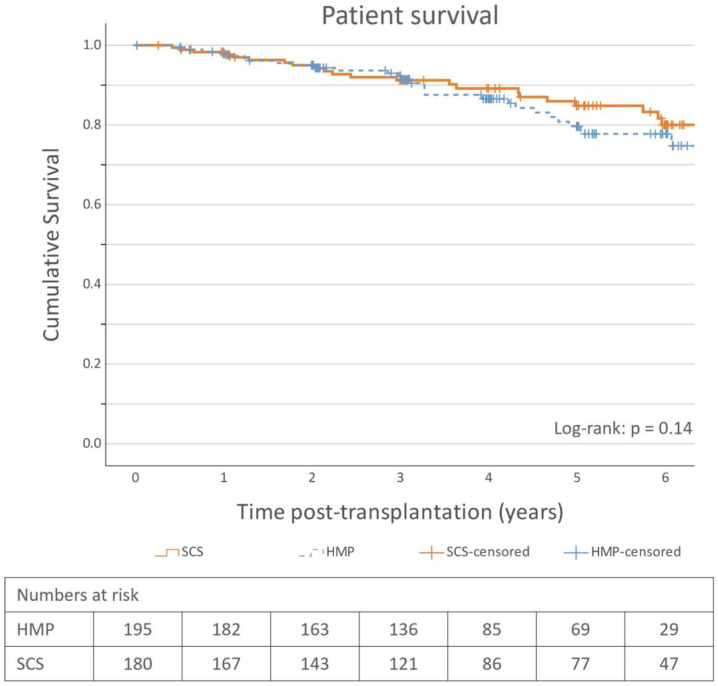
Post-transplantation patient survival in HMP and SCS groups, marginal-matched (n = 375). Numbers of deceased patients: SCS = 29; HMP = 31. HMP = hypothermic machine perfusion, SCS = static cold storage.

Survival analyses were performed using the marginal-matched cohort. There was no significant difference in cumulative incidence of graft loss (Figure 2) or patient survival (Figure 3) between the HMP and SCS groups. The 1-, 3- and 5-year graft survival rates in the HMP as compared to SCS groups were 97.4% vs. 96.7%, 94.5% vs. 95.9%, and 90.8% vs. 91.7%, respectively, and patient survival rates were 97.9% vs. 98.3%, 92.3% vs. 91.2%, and 79.7% vs. 84.8%, respectively.

The Cox proportional hazards model revealed no statistically significant hazard reduction with HMP compared to SCS for graft survival (HR = 0.79; 95% CI: 0.34–1.16; *p* = 0.51) or patient survival (HR = 0.66; 95% CI: 0.38–1.15; *p* = 0.14).

### 3.5. Graft Function

A significant benefit was observed with HMP in terms of graft function measured as eGFR at 1 month (mean difference: 4.0 [0.1; 8.0], Table 4). There was a trend for better short-term graft function at discharge and 3 months. No beneficial effect of HMP was found on graft function beyond 3 months.

### 3.6. Post-hoc Analysis

**Figure 4:**
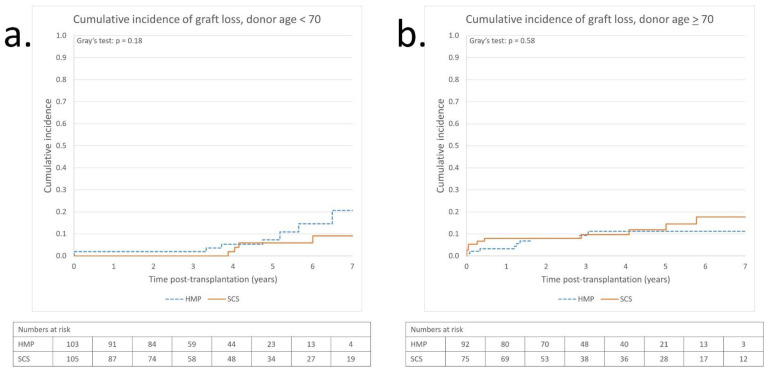
Post-transplantation cumulative incidence of graft loss in HMP and SCS groups, marginal-matched, adjusted for death as competing risk, and stratified for donor age. (**a**) Donor age < 70 (n = 208). HMP (n = 103) vs SCS (n = 105). Numbers of grafts lost: SCS = 4; HMP = 8. (**b**) Donor age > 70 (n = 167). HMP (n = 92) vs SCS (n = 75). Numbers of grafts lost: SCS = 10; HMP = 9. HMP = hypothermic machine perfusion, SCS = static cold storage.

To further investigate whether donor age affected the risk of DGF and graft survival, we performed additional tests by stratifying the participants from the marginal-matched cohort into two groups: donor age < 70 years (n = 208) and donor age ≥ 70 years (n = 167). Analysis using logistic regression and classifying donor age as a dichotomous variable (<70 and ≥70 years) to show how age influences the efficacy of preventing DGF between HMP and SCS revealed an OR of 0.43 (95% CI: 0.19–0.97) for donors < 70 years and 0.80 (95% CI: 0.28–2.24) for ≥70 years. Interaction between these age groups and HMP’s prevention of DGF was not statistically significant (*p* = 0.36). However, logistics regression adjusted for donor age as a continuous variable revealed a 46.8% risk reduction for DGF with HMP (adjusted OR = 0.53; 95% CI: 0.28–0.99; *p* = 0.045). Graft survival analysis did not show a statistically significant difference in cumulative incidence of graft loss in either age group, although a small but statistically non-significant decreased incidence of graft loss in the long-term was observed for SCS for donors aged <70 years and for HMP for donors aged ≥70 years (Figure 4a,b).

## 4. Discussion

HMP has consistently yielded better short-term results than SCS in ECD kidney transplantation, but the magnitude varies widely depending on the transplant center. However, this short-term benefit does not seem to unanimously translate into better graft function in the long term; in fact, most evidence suggests that it does not [10]. There are also limited studies comparing the two preservation methods in transplant populations with relatively high rates of graft survival, such as in Sweden [14]. Our matched retrospective study revealed a relatively low DGF-rate in both groups: 9.2% in HMP and 16.1% in SCS (Table 4). Although this difference was not statistically significant in the primary analysis (*p* = 0.063), it suggested a trend for DGF-rate reduction when using HMP. This result was strengthened by the significant benefit observed in both sensitivity analyses, suggesting that HMP may be effective in reducing DGF-rates even when the overall DGF-rate is low. With that said, it is not evident that HMP makes sense from an economic standpoint, due to the already low DGF-rates, and therefore, relatively small effect size. A follow-up study including more participants could bring more clarity. Also, cost-effectiveness and harm-benefit analyses would be of interest to further navigate the future use of HMP in ECD kidney transplantation at our center.

The trend found with HMP on DGF-rate in our study is similar to those found in studies from other centers [8,10,24]. The risk reduction for DGF of 55% with HMP (adjusted OR = 0.45, *p* = 0.012) using logistic regression adjusted for confounders in the entire cohort, as well as the logistic regression adjusted for donor age in the marginal-matched cohort (adjusted OR = 0.53, *p* = 0.045) is comparable to the adjusted OR of 0.46 reported by Treckmann et al. in their RCT in 2011 [8] and the OR of 0.485, from another matched study on donation after brain death (DBD)-kidneys by Gasteiger et al. in 2020 [25]. This comparability is interesting because the DGF-rates found in our study are at the lower end of the spectrum: 16.1% for the SCS group and 9.2% for the HMP group, compared to 29.7% and 22% reported by Treckmann et al. with similar CIT and donor age. Another Swedish study by Sedigh et al. reported a 20.3% DGF-rate in the SCS group and 16.7% in the HMP group in ECD-kidneys at a slightly shorter CIT [24]. Our DGF-rate in SCS kidneys was low compared to these studies. Interestingly, our results differ from an earlier single-center study at our center, which reported a DGF-rate of 24.1% in DBD kidneys between 2007 and 2009, of which approximately 60% were ECD [26]. The reason for the higher DGF-rates in this previous study compared to ours is not clear.

This study was not designed and did not reveal the reason for the relatively low incidence of DGF in our groups. The definition of DGF may have had an impact. DGF is mostly defined as the need for dialysis in the first week post-transplantation. Dialysis within the first 24 h post-transplantation was excluded according to the DGF definition used in our study. It is probable that using the more common definition would result in higher DGF-rates and better comparisons with other studies. In a single-center UK-based study investigating the effect of different DGF-definitions on the outcomes, DGF-rates using our definition reached 32.4%, compared to 40.0% when using the more traditional definition in a DBD cohort [27]. Therefore, we expect our DGF-rates to be underestimated only marginally and the overall DGF-rates to remain relatively low. If the different treatment arms were affected differently, there is a possibility that this may have affected the outcome. Unfortunately, there are no studies to our knowledge that have investigated this.

Our marginal-matching procedure yielded an SCS group that was 2.5 years older (mean) and had 1 h longer CIT (mean and median) than the unmatched cohort. Matching also resulted in a more comparable KDPI between the groups, which was favorable in our graft survival analyses. Donor age and CIT are important risk factors for DGF, and we assumed increasing these would increase the DGF-rates [28]. Surprisingly, this was not the case; instead, the DGF-rate decreased slightly from 17.3% to 16.1% after matching. We used only donor age, CIT, and graft number as matching variables, and although they are important, there is a possibility that other risk factors were unevenly excluded in our matching procedure, causing this small decrease in the DGF-rate, yielding the main analysis insignificant. However, there was no obvious skewness comparing baseline characteristics before and after matching (Table 2 and Table 3) as SMD was consistently very low and no difference between the groups reached significance, except for donor terminal creatinine and eGFR. Although our collection of risk factors was extensive, the possibility remains that unmeasured variables were significantly different between the groups, influencing the risk of DGF. Nevertheless, because of the similarities between the groups after matching and the similar efficacy of DGF reduction in the primary and sensitivity analyses, we are confident in our matching and the results.

Our post-hoc interaction analysis, investigating whether the effect of HMP on DGF varies with donor age, did not show a statistically significant interaction (*p* = 0.36) or better effect in any age group, although there was an indication of a better effect for donors aged <70 years (OR 0.43) than for those aged ≥70 (OR 0.80). However, because of the relatively few cases of DGF and participants in these groups, these analyses have low power. This is also true in the survival analyses (Figure 4a,b). Hence, studies with a greater number of participants are needed to draw any firm conclusions on whether HMP is more effective in kidneys donated by younger or older donors.

The overall graft survival rates were high (>90% at 5 years) in both the SCS and HMP groups, and there was no apparent survival benefit using HMP (Figure 2). The most remarkable finding was the relatively high graft survival rate, especially in the SCS group (91.7% at 5-years). This is comparable to the results presented by other studies on Swedish populations. The SNR in their 2022 annual report reported similar survival rates: 93.3% at 5 years for kidneys from deceased donors [14]. Another study of kidney transplantation in Sweden between 2005 and 2013, regardless of the donor type, reported 1-year and 5-year death-censored graft survival rates of 97.9% and 94.2%, respectively [15], which are consistent with our results. This is relatively high compared to international studies in the last decade. For SCS, studies have reported survival rates of 70–97% at 1-year, 80–85% at 3 years, and approximately 80% at 5 years [10,29,30]. The previously mentioned international landmark RCT performed in Western European countries within Eurotransplant reported 1- and 3-year graft survival rates of 80% and 76%, respectively, in their SCS subgroup for ECD kidneys and a statistically significant survival benefit using HMP [8,9]. In contrast, a large French study found no graft survival benefit using HMP at 5 years [31]. There are limited data on long-term graft survival comparing HMP to SCS, and evidence for the benefit of using HMP is weak, which is also corroborated by our study. The cumulative incidence of graft loss, and subsequently the graft survival rates, was comparable between the groups, and no conclusion can be made regarding whether HMP affects graft survival with the dataset used in the study. This is also the case for patient survival, as no significant patient survival benefit was observed.

The reason for the good outcomes observed in Sweden is relatively unknown. Donor-related factors do not seem to explain it, indeed the KDPI was relatively high. It is therefore likely that several other factors, such as recipient-related factors, universal and equitable health coverage, organ allocation system, access to transplantation and immunosuppressive medications, good follow-up regimens as well as national health quality registries in Sweden that provide a unique opportunity to monitor quality and results may contribute. Unfortunately, this is mere speculation; to our knowledge, no studies have been conducted comparing these processes to other countries.

The post-transplantation graft function was similar between the groups. There was a trend, although very modest, of initially better graft function in the HMP group, although it only reached significance at 1 month (Table 4). This could be due to less damage from the transplantation process and, therefore, quicker return of kidney function using HMP. However, since the difference was very small it should be interpreted with caution. Also, no long-term benefit was observed on kidney function, in accordance with current knowledge.

This study had a few limitations. The most obvious is the retrospective and non-randomized nature of the study, and although we performed a matching of good quality, the results can never reach the strength of an RCT. There are also several potential biases. As previously discussed, our DGF-definition, excluding recipients receiving dialysis the first 24 h post-transplantation may complicate comparisons to other studies. The sample size was also small to confidently reveal long-term differences between the groups, especially in the stratified analyses. The inclusion of SCS-kidneys in 2013 may have induced a time-period bias, although this is expected to be limited. The selection of the preservation method was not randomized, and although every ECD-kidney should receive HMP, this was not the case in 186 kidneys between 2014 and 2021. Whether this was due to logistical, donor, or recipient factors, there is potential for selection bias, although we tried to mitigate this to some extent in our matching procedures. Matching may cause clustering or dispersion of confounding factors not included in the matching model; however, we did not find any obvious disproportions of important variables due to matching and the robustness of the matching was also evaluated using our sensitivity analyses, suggesting adequate matching parameters. The results of this study are from a single center in Sweden and cannot be generalized to other populations.

The strengths of the study include an extensive and well-characterized cohort, with a low number of dropouts, and the use of the matching procedure that generated comparable groups of important risk factors for both DGF and graft survival, thus strengthening the results. The inclusion of more baseline characteristics in a multivariable analysis including all the participants was also performed to gauge their effect on the endpoints and support the results. We also used the cumulative incidence function for a more correct and accurate graft survival analysis [22]. This being a single-center study, the comparability between the groups may be better, as it is likely that kidney transplantation and preservation procedures, DGF diagnostics, and immunosuppressive therapy are very similar.

## 5. Conclusions

In this study, we sought to investigate the efficacy of HMP in ECD kidney transplantation at our center. There was an overall relatively low rate of DGF. HMP showed a trend to lower DGF-rate in the marginal-matched comparison, which was strengthened by the significant benefit found in the sensitivity analyses, suggesting a possible short-term benefit of kidney function using HMP. No long-term benefits were observed for graft survival or kidney function.

## Figures and Tables

**Table 1:** Primary and secondary endpoints.

Primary Endpoint	Definition
Delayed graft function (y/n)	Need for dialysis within first week post-Tx, excluding first 24 h
**Secondary endpoints**	**Definition**
Graft survival at 1-, 3-, and 5-years	Graft loss defined as return to dialysis or re-Tx. Calculated as 1 minus the cumulative incidence at the time of interest.
Patient survival at 1-, 3-, and 5-years	
DGF-duration (days)	Duration between Tx and initiation of graft function if DGF occurred
Primary-non function (y/n)	Failure of graft to ever function
Early graft loss (y/n)	Graft loss within first 30 days post-Tx
Length of hospitalization (days)	
Rejection reason for graft loss (y/n)	Acute or chronic rejection on biopsy prior to graft loss
Renal function at discharge, 1, 3, 6, 12-, 24-, 36-, and 60-months post transplantation (eGFR) ^†^	

DGF = Delayed graft function; eGFR = estimated glomerular filtration rate; Tx = Transplantation. ^†^ Calculated based on S/P-creatinine using the Chronic Kidney Disease Epidemiology Collaboration (CDK-EPI) 2009 equation [20].

**Table 2:** Clinical characteristics at baseline of the whole cohort (prior to matching).

	HMP (n = 195)	SCS (n = 243)	*p*-Value	SMD
Donors				
Age (years)	68.5 (7.1)	65.7 (7.2)	0.0002	0.386
	69.0 (64.0; 73.0)	66.0 (61.0; 71.0)		
CIT (hours)	14.4 (4.2)	12.8 (4.3)	<0.0001	0.368
	14.1 (11.4; 17.5)	12.9 (9.7; 15.6)		
KDPI (%)	87.8 (10.7)	83.6 (11.9)	0.0003	0.375
	90.0 (83.0; 96.0)	85.0 (76.0; 94.0)		
BMI	26.3 (4.4)	26.0 (4.3)	0.42	0.078
	26.1 (23.4; 28.7)	25.3 (23.1; 28.4)		
Terminal serum/plasma creatinine (µmol/L)	92.6 (56.3)	79.7 (37.4)	0.004	0.277
	78.0 (56.0; 101.0)	73.0 (56.0; 90.0)		
Terminal eGFR (mL/min/1.73 m^2^)	74.3 (24.5)	82.2 (21.2)	0.0006	0.346
	78.7 (57.6; 93.5)	88.5 (67.4; 97.1)		
Male gender	113 (57.9%)	138 (56.8%)	0.88	0.02
History of hypertension	108 (55.4%)	124 (51.0%)	0.42	0.09
History of diabetes ^†^	13 (6.7%)	23 (9.5%)	0.41	0.10
Cerebrovascular cause of death	132 (67.7%)	181 (74.5%)	0.17	0.14
Hepatitis C positive	2 (1.0%)	0 (0.0%)	0.40	0.14
**Recipients**				
Age (years)	60.9 (9.2)	59.0 (10.2)	0.052	0.188
	62.0 (55.0; 69.0)	60.0 (52.0; 67.0)		
Male gender	119 (61.0%)	158 (65.0%)	0.45	0.08
First kidney graft	166 (85.1%)	209 (86.0%)	0.90	0.03
Duration of dialysis (months) ^‡^	27.9 (23.1)	28.9 (24.5)	0.67	0.042
	24.0 (11.0; 40.0)	26.0 (9.0; 42.0)		
Kidney disease				
*Glomerular disease*	52 (26.7%)	57 (23.5%)	0.51	0.07
*Vasculitis*	4 (2.1%)	4 (1.6%)	1.00	0.03
*Interstitial nephritis or pyelonephritis*	9 (4.6%)	18 (7.4%)	0.31	0.12
*Diabetic nephropathy*	19 (9.7%)	34 (14.0%)	0.23	0.13
*Hypertensive nephrosclerosis*	19 (9.7%)	18 (7.4%)	0.48	0.08
*Polycystic kidney disease (ADPKD)*	40 (20.5%)	52 (21.4%)	0.92	0.02
*Hereditary or congenital renal disease*	7 (3.6%)	11 (4.5%)	0.81	0.05
*Other*	43 (22.1%)	49 (20.2%)	0.71	0.05
Comorbidities				
*Hypertension*	170 (87.2%)	210 (86.4%)	0.93	0.02
*Cardiovascular disease*	48 (24.6%)	54 (22.2%)	0.63	0.06
*Cerebrovascular disease*	16 (8.2%)	13 (5.3%)	0.32	0.11
*Diabetes*	18 (9.2%)	34 (14.0%)	0.17	0.15
*Thromboembolic disease*	8 (4.1%)	12 (4.9%)	0.86	0.04
*Lung disease*	15 (7.7%)	19 (7.8%)	1.00	0.00
*Hepatic or Gastrointestinal disease*	37 (19.0%)	42 (17.3%)	0.74	0.04
*History of malignancy*	22 (11.3%)	29 (11.9%)	0.95	0.02
*Other*	105 (53.8%)	142 (58.4%)	0.39	0.09
PRA Class 1 (%)	5.4 (16.3)	8.6 (23.1)	0.10	0.157
	0 (0; 0)	0 (0; 0)		
PRA Class 2 (%)	8.56 (23.5)	10.1 (25.4)	0.54	0.061
	0 (0; 0)	0 (0; 0)		
HLA-mismatch: HLA-A, -B, -DR (0–6)	3.97 (1.40)	3.83 (1.62)	0.35	0.094
	4 (3; 5)	4 (3; 5)		
HLA-DQ mismatch (0–2) ^§^	0.65 (0.64)	0.65 (0.59)	1.00	0.008
	1 (0; 1)	1 (0; 1		
CMV-mismatch	32 (16.4%)	30 (12.3%)	0.28	0.12

Data are presented as mean (SD), median (Q1; Q3), or number (percentage).Abbreviations: HMP, hypothermic machine perfusion; SCS, static cold storage; n, number of grafts; SMD, standardized mean difference; CIT, cold ischemia time; KDPI, kidney donor profile index; BMI, Body mass index; eGFR, estimated glomerular filtration rate; PRA, panel reactive antibodies; HLA, Human leukocyte antigen; CMV, cytomegalovirus. Continuous variables were tested for significance using Fisher’s Non-Parametric Permutation Test. Binary variables with Fisher’s exact test. ^†^ 4 missing data in the HMP group. ^‡^ 5 missing in the SCS group and 2 in the HMP group. ^§^ 14 missing in the SCS group and 3 in the HMP group.

**Table 3:** Clinical characteristics at baseline of marginal-matched cohort based on propensity score.

	HMP (n = 195)	SCS (n = 180)	*p*-Value	SMD
Donors				
Age (years)	68.5 (7.1)	68.1 (6.1)	0.6	0.058
	69.0 (64.0; 73.0)	68.0 (64.0; 72.0)		
CIT (hours)	14.4 (4.2)	13.8 (4.2)	0.21	0.129
	14.1 (11.4; 17.5)	13.8 (10.8; 16.5)		
KDPI (%)	87.8 (10.7)	86.4 (10.6)	0.20	0.132
	90.0 (83.0; 96.0)	89.0 (78.0; 96.0)		
BMI	26.3 (4.40)	25.9 (3.96)	0.36	0.095
	26.1 (23.4; 28.7)	25.3 (23.3; 28.2)		
Terminal serum/plasma creatinine (µmol/L)	92.6 (56.3)	79.8 (38.2)	0.01	0.264
	78.0 (56.0; 101.0)	73.0 (55.0; 89.0)		
Terminal eGFR (mL/min/1.73 m^2^)	74.3 (24.5)	81.1 (20.8)	0.004	0.296
	78.7 (57.6; 93.5)	87.3 (67.0; 95.4)		
Male gender	113 (57.9%)	103 (57.2%)	0.97	0.01
History of hypertension	108 (55.4%)	86 (47.8%)	0.17	0.15
History of diabetes ^†^	13 (6.7%)	17 (9.4%)	0.46	0.10
Cerebrovascular cause of death	132 (67.7%)	132 (73.3%)	0.28	0.12
Hepatitis C positive	2 (1.0%)	0 (0.0%)	0.54	0.14
**Recipients**				
Age (years)	60.9 (9.2)	60 (9.3)	0.35	0.098
	62.0 (55.0; 69.0)	60.0 (53.5; 67.0)		
Male gender	119 (61.0%)	116 (64.4%)	0.56	0.07
First kidney graft	166 (85.1%)	153 (85.0%)	0.90	0.00
Duration of dialysis (months) ^‡^	27.9 (23.1)	28.5 (23.9)	0.81	0.025
	24.0 (11.0; 40.0)	25.0 (9.5; 41.5)		
Kidney disease				
*Glomerular disease*	52 (26.7%)	43 (23.9%)	0.62	0.06
*Vasculitis*	4 (2.1%)	4 (2.2%)	1.00	0.01
*Interstitial nephritis or pyelonephritis*	9 (4.6%)	14 (7.8%)	0.29	0.13
*Diabetic nephropathy*	19 (9.7%)	25 (13.9%)	0.28	0.13
*Hypertensive nephrosclerosis*	19 (9.7%)	14 (7.8%)	0.63	0.07
*Polycystic kidney disease (ADPKD)*	40 (20.5%)	34 (18.9%)	0.79	0.04
*Hereditary or congenital renal disease*	7 (3.6%)	7 (3.9%)	1.00	0.02
*Other*	43 (22.1%)	39 (21.7%)	1.00	0.01
Comorbidities				
*Hypertension*	170 (87.2%)	157 (87.2%)	1.00	0.00
*Cardiovascular disease*	48 (24.6%)	41 (22.8%)	0.77	0.04
*Cerebrovascular disease*	16 (8.2%)	9 (5.0%)	0.30	0.13
*Diabetes*	18 (9.2%)	28 (15.6%)	0.087	0.19
*Thromboembolic disease*	8 (4.1%)	7 (3.9%)	1.00	0.01
*Lung disease*	15 (7.7%)	12 (6.7%)	0.86	0.04
*Hepatic or Gastrointestinal disease*	37 (19.0%)	29 (16.1%)	0.55	0.08
*History of malignancy*	22 (11.3%)	22 (12.2%)	0.90	0.03
*Other*	105 (53.8%)	110 (61.1%)	0.19	0.15
PRA Class 1 (%)	5.4 (16.3)	8.5 (23.6)	0.15	0.152
	0 (0; 0)	0 (0; 0)		
PRA Class 2 (%)	8.6 (23.5)	10.6 (26.4)	0.43	0.084
	0 (0; 0)	0 (0; 0)		
HLA-mismatch: HLA-A, -B, -DR (0–6)	3.97 (1.40)	3.84 (1.68)	0.43	0.088
	4 (3; 5)	4 (3; 5)		
HLA-DQ mismatch (0–2) ^§^	0.65 (0.64)	0.67 (0.61)	0.85	0.028
	1 (0; 1)	1 (0; 1)		
CMV-mismatch	32 (16.4%)	24 (13.3%)	0.49	0.09

Data are presented as mean (SD), median (Q1; Q3), or number (percentage). Abbreviations: HMP, hypothermic machine perfusion; SCS, static cold storage; n, number of grafts; SMD, standardized mean difference; CIT, cold ischemia time; KDPI, kidney donor profile index; BMI, Body mass index; eGFR, estimated glomerular filtration rate; PRA, panel reactive antibodies; HLA, Human leukocyte antigen; CMV, cytomegalovirus. Continuous variables were tested for significance using Fisher’s Non-Parametric Permutation Test. Binary variables with Fisher’s exact test. ^†^ 4 missing in the HMP group. ^‡^ 4 missing in the SCS group and 2 in the HMP group. ^§^ 14 missing in the SCS group and 3 in the HMP group.

**Table 4:** Analyses of the outcome variables between HMP and SCS groups, marginal-matched on the propensity score. Primary analysis.

	HMP (n = 195)	SCS (n = 180)	Mean Difference (95% CI)	Effect Size	*p*-Value
Primary endpoint					
DGF	18 (9.2%)	29 (16.1%)	−6.9 (−14.1; 0.4)	0.21	0.063
**Secondary endpoints**					
PNF	1 (0.5%)	2 (1.1%)	−0.6 (−3.0; 1.8)	0.07	0.94
Early graft loss	4 (2.1%)	4 (2.2%)	−0.2 (−3.6; 3.3)	0.01	1.00
Rejection reason for graft loss ^†^	3/16 (18.8%)	3/12 (27.3%)	−8.5 (−44.7; 26.5)	0.20	0.94
DGF duration (days)	10.8 (10.8)	11.5 (9.6)	−0.7 (−7.2; 5.1)	0.08	0.84
	8.0 (4.0; 13.0)	9.0 (6.0; 13.0)			
Length of hospitalization (days)	7.8 (7.7)	7.2 (4.6)	0.6 (−0.7; 1.9)	0.09	0.38
	5.0 (5.0; 8.0)	6.0 (5.0; 8.0)			
eGFR (mL/min/1.73 m^2^)					
*At discharge*	28.7 (19.9)	25.4 (17.3)	3.3 (−0.5; 7.1)	0.18	0.091
	25.5 (12.2; 38.4)	19.7 (11.7; 36.2)			
*1 month*	44.5 (20.7)	40.4 (13.9)	4.1 (0.1; 8.0)	0.23	0.044
	41.9 (29.1; 55.8)	39.5 (31.4; 49.4)			
*3 months*	45.9 (18.0)	42.9 (13.8)	3.0 (−0.3; 6.4)	0.19	0.078
	44.4 (32.7; 55.4)	40.5 (33.7; 51.1)			
*6 months*	44.8 (18.0)	43.8 (14.6)	1.1 (−2.4; 4.6)	0.07	0.55
	43.5 (30.3; 53.6)	42.4 (34.0; 51.6)			
*12 months*	45.8 (17.7)	44.4 (14.1)	1.4 (−2.0; 4.9)	0.09	0.43
	44.6 (33.3; 55.4)	43 (34.5; 53.0)			
*24 months*	45.6 (18.4)	42.5 (15.0)	3.1 (−0.9; 7.0)	0.18	0.12
	43.6 (32.9; 55.8)	41.3 (31.5; 52.9)			
*36 months*	42.7 (17.1)	42.7 (15.3)	0.0 (−4.0; 4.0)	0.00	0.99
	41.3 (31.1; 53.1)	41.7 (32.7; 51.8)			
*60 months*	42.2 (19.0)	42.9 (17.2)	−0.7 (−6.6; 5.5)	0.04	0.83
	40.2 (29.8; 57.0)	42.7 (33.7; 53.7)			

Data are presented as mean (SD), median (Q1; Q3), or number (percentage). Abbreviations: HMP, hypothermic machine perfusion; SCS, static cold storage; DGF, delayed graft function; PNF, primary non-function; eGFR, estimated glomerular filtration rate. The effect size is the absolute difference in the mean divided by the pooled SD. Continuous variables were tested for significance using Fisher’s Non-Parametric Permutation Test. Binary variables with Fisher’s exact test. ^†^ Only death-adjusted graft loss in the analysis. Presented as the total number and percentage of reasons for graft loss.

**Table 5:** Multivariable logistic regression analyses on the entire cohort (prior to matching) adjusted for multiple confounders. First sensitivity analysis.

	HMP (n = 195)	SCS (n = 243)		*p*-Value
**Primary endpoint**			**Adjusted OR (95% CI)**	
DGF	18 (9.2%)	29 (16.1%)	0.45 (0.24; 0.84)	0.012
**Secondary endpoints**			**Adjusted mean difference (95% CI)**	
DGF duration (days)	11.1	19.1	−7.9 (−35.5; 19.5)	0.56
Length of hospitalization (days)	7.9	7.8	0.1 (−1.2; 1.4)	0.91
eGFR (mL/min/1.73 m^2^)				
*At discharge*	31.3	26.1	5.2 (1.7; 8.6)	0.004
*1 month*	46.2	41.8	4.4 (0.9; 7.9)	0.014
*3 months*	47.3	43.4	3.9 (0.9; 6.9)	0.011
*6 months*	46.3	44.3	2.0 (−1.0; 5.1)	0.19
*12 months*	47.1	45.0	2.1 (−1.0; 5.2)	0.19
*24 months*	47.2	43.1	4.1 (0.5; 7.7)	0.025
*36 months*	44.1	43.5	0.6 (−3.2; 4.4)	0.75
*60 months*	43.7	44.5	−0.8 (−6.2; 4.5)	0.76

Data are presented as adjusted mean or number (percentage). Adjusted for donor and recipient age, cold ischemia time, graft number, donor terminal creatinine and terminal eGFR. Binary variables analyzed using multivariable logistic regression, presented as adjusted OR, and continuous variables using analysis of covariance (ANCOVA), presented as adjusted mean difference. Primary non-function, early graft loss, and rejection reason for graft loss not included due to too few events. Abbreviations: HMP, hypothermic machine perfusion; SCS, static cold storage; DGF, delayed graft function; OR, odds ratio; eGFR, estimated glomerular filtration rate.

## Data Availability

The data presented in this study are available upon reasonable request. The data are not publicly available due to data protection and ethical restrictions.

## Appendix A

### Appendix A.1. Matching Methods

Marginal distribution matching is a group-level matching. The participants in the pool of SCS kidneys were selected individually, taking the individual in the SCS group that minimized the maximum t-value between the groups regarding donor age, CIT, and number of grafts. This was performed until no more controls could be included without making the groups too dissimilar.

Caliper matching is an ID level-matching method [32]. For each subject in the test group, a control subject was identified using the following caliper widths: 8 years for donor age, 1.5 h for CIT, and exact match for graft number.

The propensity score is the probability of assignment to a treatment group based on baseline characteristics and can be used for matching [33]. We generated the propensity score with logistic regression using HMP as the dependent variable and confounding variables (donator age, cold ischemia time, recipient age, and graft number) as independent variables. Propensity score can also be used in combination with other matching methods. We used marginal distribution matching on the propensity score on logit (prop.score) = ln(prop.score)/(1-prop.score), which generated the best matched group used in the primary analysis.

### Appendix A.2. Tables

**Table A1:** Clinical characteristics at baseline of the ID-matched groups.

	HMP (n = 172)	SCS (n = 172)	*p*-Value	SMD
Donors				
Age (years)	67.5 (6.7)	66.9 (6.4)	0.36	0.099
	68.0 (63.0; 72.0)	66.0 (62.0; 72.0)		
CIT (hours)	13.7 (3.8)	13.7 (3.8)	0.98	0.002
	13.6 (11.0; 16.4)	13.7 (11.1; 16.4)		
KDPI	87.2 (10.9)	84.9 (11.2)	0.056	0.208
	90.0 (82.5; 96.0)	87.0 (77.0; 95.0)		
BMI	26.4 (4.52)	26.0 (4.26)	0.35	0.099
	26.1 (23.5; 28.7)	25.3 (23.2; 28.6)		
Terminal S/P-Creatinine	94.6 (58.6)	80.5 (38.6)	0.008	0.285
	79.0 (60.0; 104.0)	74.5 (56.0; 89.5)		
Terminal eGFR	73.6 (25.1)	81.5 (21.8)	0.002	0.337
	78.3 (56.9; 93.3)	87.9 (66.6; 97.1)		
Male gender	95 (55.2%)	101 (58.7%)	0.59	0.07
History of hypertension	95 (55.2%)	85 (49.4%)	0.33	0.12
History of diabetes ^†^	12 (7.0%)	19 (11.0%)	0.29	0.14
Cerebrovascular cause of death	117 (68.0%)	123 (71.5%)	0.56	0.08
Hepatitis C positive	2 (1.2%)	0 (0.0%)	0.50	0.15
**Recipients**				
Age (years)	60.0 (9.3)	59.5 (9.6)	0.65	0.05
	61.0 (54.0; 68.0)	60.0 (53.0; 68.0)		
Male gender	107 (62.2%)	114 (66.3%)	0.50	0.08
First kidney graft	151 (87.8%)	151 (87.8%)	0.90	0.00
Duration of dialysis (months) ^‡^	28.2 (23.3)	29.9 (23.7)	0.52	0.069
	24.1 (11.0; 40.0)	26.0 (10.0; 43.0)		
Kidney disease				
*Glomerular disease*	43 (25.0%)	41 (23.8%)	0.90	0.03
*Vasculitis*	4 (2.3%)	4 (2.3%)	1.00	0.00
*Interstitial nephritis or pyelonephritis*	9 (5.2%)	13 (7.6%)	0.51	0.10
*Diabetic nephropathy*	18 (10.5%)	24 (14.0%)	0.41	0.11
*Hypertensive nephrosclerosis*	15 (8.7%)	13 (7.6%)	0.84	0.04
*Polycystic kidney disease (ADPKD)*	34 (19.8%)	40 (23.3%)	0.51	0.08
*Hereditary or congenital renal disease*	6 (3.5%)	7 (4.1%)	1.00	0.03
*Other*	41 (23.8%)	30 (17.4%)	0.18	0.16
Comorbidities				
*Hypertension*	153 (89.0%)	148 (86.0%)	0.51	0.09
*Cardiovascular disease*	40 (23.3%)	38 (22.1%)	0.90	0.03
*Cerebrovascular disease*	14 (8.1%)	9 (5.2%)	0.39	0.12
*Diabetes*	16 (9.3%)	26 (15.1%)	0.14	0.18
*Thromboembolic disease*	7 (4.1%)	10 (5.8%)	0.62	0.08
*Lung disease*	13 (7.6%)	11 (6.4%)	0.83	0.05
*Hepatic or Gastrointestinal disease*	30 (17.4%)	31 (18.0%)	1.00	0.02
*History of malignancy*	18 (10.5%)	20 (11.6%)	0.86	0.04
*Other*	90 (52.3%)	102 (59.3%)	0.23	0.14
PRA Class 1 (%)	8.3 (23.0)	9.6 (24.5)	0.10	0.180
	0 (0; 0)	0 (0; 0)		
PRA Class 2 (%)	8.3 (23.0)	9.9 (24.7)	0.54	0.066
	0 (0; 0)	0 (0; 0)		
HLA-mismatch: HLA-A, -B, -DR (0–6)	4.03 (1.37)	3.73 (1.66)	0.077	0.195
	4 (3; 5)	4 (3; 5)		
HLA-DQ mismatch (0–2) ^§^	0.66 (0,66)	0.66 (0.60)	0.98	0.012
	1 (0; 1)	1 (0; 1)		
CMV-mismatch	31 (18.0%)	21 (12.2%)	0.18	0.16

Data are presented as mean (SD), median (Q1; Q3), or number (percentage). Caliper widths: Donor age 8 years; CIT 1.5h; graft number exact match. Abbreviations: HMP, hypothermic machine perfusion; SCS, static cold storage; n, number of grafts; SMD, standardized mean difference; CIT, cold ischemia time; KDPI, kidney donor profile index; BMI, Body mass index; eGFR, estimated glomerular filtration rate; PRA, panel reactive antibodies; HLA, Human leukocyte antigen; CMV, cytomegalovirus. Continuous variables were tested for significance using Fisher’s Non-Parametric Permutation Test. Binary variables with Fisher’s exact test. ^†^ 4 missing data in the HMP group. ^‡^ 1 missing data in the SCS group and 2 in the HMP group. ^§^ 14 missing data in the SCS group and 3 in the HMP group.

**Table A2:** Analyses of the outcomes between HMP and SCS groups, ID-matched. Second sensitivity analysis.

	HMP (n = 172)	SCS (n = 172)	Mean Difference (95% CI)	Effect Size	*p*-Value
**Primary endpoint**					
DGF	15 (8.7%)	30 (17.4%)	−8.7 (−16.4; −1.1)	0.26	0.024
**Secondary endpoints**					
PNF	1 (0.6%)	2 (1.2%)	−0.6 (−3.1; 2.0)	0.06	1.00
Early graft loss	3 (1.7%)	2 (1.2%)	0.6 (−2.5; 3.7)		1.00
Rejection reason for graft loss ^†^	2/12 (16.7%)	3/9 (33.3%)	−16.7 (−56.2; 23.2)	0.39	0.71
DGF duration (days)	8.9 (6.41)	22.5 (59.9)	−13.6 (−45.9; 2.4)	0.274	0.27
	8.0 (3.0; 13.0)	9.0 (6.0; 17.0)			
Length of hospitalization (days)	7.4 (7.0)	7.6 (5.3)	−0.2 (−1.5; 1.1)	0.025	0.83
	5.0 (5.0; 8.0)	6.0 (5.0; 8.0)			
eGFR (mL/min/1.73 m^2^)					
*At discharge*	29.3 (19.5)	25.0 (17.4)	4.3 (0.4; 8.2)	0.232	0.031
	25.8 (13.5; 38.4)	19.4 (11.2; 36.2)			
*1 month*	45.0 (20.6)	40.0 (15.3)	5.0 (0.8; 9.2)	0.277	0.019
	42.2 (29.8; 55.8)	39.0 (29.3; 48.5)			
*3 months*	47.0 (17.3)	43.0 (14.3)	4.0 (0.6; 7.5)	0.251	0.023
	45.0 (33.4; 55.6)	40.8 (33.6; 52.1)			
*6 months*	45.9 (17.6)	44.0 (15.0)	1.9 (−1.7; 5.4)	0.115	0.31
	45.1 (31.3; 53.8)	42.3 (33.9; 53.5)			
*12 months*	46.6 (17.3)	44.1 (14.3)	2.5 (−1.0; 6.1)	0.157	0.17
	46.1 (33.7; 56.1)	42.9 (34.5; 53.4)			
*24 months*	47.0 (18.2)	42.6 (15.3)	4.4 (0.2; 8.4)	0.258	0.039
	44.8 (34.0; 57.4)	40.4 (32.1; 52.9)			
*36 months*	43.3 (17.0)	43.0 (16.1)	0.2 (−3.9; 4.5)	0.013	0.92
	43.0 (32.7; 53.5)	41.2 (31.3; 54.2)			
*60 months*	42.7 (19.1)	42.7 (17.0)	−0.02 (−6.2; 6.2)	0.001	0.99
	40.8 (33.2; 52.7)	40.9 (31.1; 57.2)			

Data are presented as mean (SD), median (Q1; Q3), or number (percentage). Caliper widths: Donor age 8 years; CIT 1.5 h; graft number exact match. Abbreviations: HMP, hypothermic machine perfusion; SCS, static cold storage; DGF, delayed graft function; PNF, primary non-function; eGFR, estimated glomerular filtration rate. The effect size is the absolute difference in the mean divided by the pooled SD. Continuous variables were tested for significance using Fisher’s Non-Parametric Permutation Test. Binary variables with Fisher’s exact test. ^†^ Only death-adjusted graft loss in the analysis. Presented as the total number and percentage of reasons for graft loss.

**Table A3:** Univariable paired differences between HMP and SCS groups, ID-matched. Second sensitivity analysis.

Variable	HMP Yes SCS No	HMP and SCS Equal	HMP No SCS Yes	*p*-Value
DGF	9 (5.2%)	139 (80.8%)	24 (14.0%)	0.014

Caliper widths: Donor age 8 years; cold ischemia time 1.5 h; graft number exact match. Data are presented as number (percentage). Abbreviations: HMP, hypothermic machine perfusion; SCS, static cold storage; DGF, delayed graft function. Significance was tested using Sign test.

**Table A4:** Analyses of the outcomes between HMP and SCS groups of the whole cohort (prior to matching). Complementary analysis.

	HMP (n = 195)	SCS (n = 243)	Mean Difference (95% CI)	Effect Size	*p*-Value
**Primary endpoint**					
DGF	18 (9.2%)	42 (17.3%)	−8.1 (−14.8; −1.3)	0.24	0.020
**Secondary endpoints**					
PNF	1 (0.5%)	2 (0.8%)	−0.3 (−2.3; 1.7)	0.04	1.00
Early graft loss	4 (2.1%)	4 (1.6%)	0.4 (−2.6; 3.4)	0.03	1.00
Rejection reason for graft loss ^†^	3/16 (18.8%)	5/15 (33.3%)	−14.6 (−45.7; 17.8)	0.34	0.61
DGF duration (days)	10.8 (10.8)	19.2 (50.7)	−8.43 (−34.10; 4.55)	0.195	0.61
	8.0 (4.0; 13.0)	9.0 (6.0; 17.0)			
Length of hospitalization (days)	7.8 (7.7)	7.83 (6.0)	−0.036 (−1.32; 1.23)	0.005	0.97
	5.0 (5.0; 8.0)	6.0 (5.0; 8.0)			
eGFR (mL/min/1.73 m^2^)					
*At discharge*	28.7 (19.9)	28.2 (18.4)	0.46 (−3.17; 4.08)	0.024	0.81
	25.5 (12.2; 38.4)	23.0 (12.5; 41.7)			
*1 month*	44.5 (20.7)	43.2 (15.6)	1.25 (−2.57; 5.04)	0.069	0.52
	41.9 (29.1; 55.8)	41.8 (32.7; 53.3)			
*3 months*	45.9 (18.0)	44.6 (14.8)	1.3 (−1.9; 4.5)	0.079	0.43
	44.4 (32.7; 55.4)	41.9 (35.7; 53.4)			
*6 months*	44.8 (18.0)	45.4 (15.1)	−0.6 (−3.8; 2.6)	0.036	0.73
	43.5 (30.3; 53.5)	44.7 (35.2; 54.3)			
*12 months*	45.8 (17.7)	46.0 (14.5)	−0.2 (−3.5; 3.0)	0.015	0.90
	44.6 (33.3; 55.4)	44.1 (35.0; 55.2)			
*24 months*	45.6 (18.4)	44.4 (15.4)	1.2 (−2.5; 4.9)	0.073	0.52
	43.6 (32.9; 55.8)	43.1 (33.1; 56.3)			
*36 months*	42.7 (17.1)	44.7 (16.0)	−2.0 (−5.8; 1.8)	0.12	0.29
	41.3 (31.1; 53.1)	43.5 (33.0; 56.1)			
*60 months*	42.2 (19.0)	45.5 (16.8)	−3.3 (−8.8; 2.2)	0.184	0.24
	40.2 (29.8; 57.0)	45.1 (34.4; 56.8)			

Data are presented as mean (SD), median (Q1; Q3), or number (percentage). Abbreviations: HMP = hypothermic machine perfusion, SCS = static cold storage, DGF = delayed graft function, PNF = primary non-function, eGFR = estimated glomerular filtration rate.The effect size is the absolute difference in the mean divided by the pooled SD. Continuous variables were tested for significance using Fisher’s Non-Parametric Permutation Test. Binary variables with Fisher’s exact test. † Only death-adjusted graft loss in the analysis. Presented as the total number and percentage of reasons for graft loss.
